# Advances in Functional Imaging of Differentiated Thyroid Cancer

**DOI:** 10.3390/cancers13194748

**Published:** 2021-09-23

**Authors:** Michele Klain, Emilia Zampella, Carmela Nappi, Emanuele Nicolai, Raffaele Ambrosio, Elena Califaretti, Livia Lamartina, Martin Schlumberger, Désirée Deandreis, Domenico Salvatore, Alberto Cuocolo

**Affiliations:** 1 Department of Advanced Biomedical Sciences, University of Naples “Federico II”, 80131 Naples, Italy; micheleklain@libero.it (M.K.); emilia.zampella@gmail.com (E.Z.); c.nappi@unina.it (C.N.); 2 IRCCS SDN, 80143 Naples, Italy; emanuele.nicolai@synlab.it (E.N.); ambrosioraf@libero.it (R.A.); 3 Department of Medical Science, Nuclear Medicine Division, University of Turin, 80126 Torino, Italy; elena.califaretti@unito.it (E.C.); desiree.deandreis@unito.it (D.D.); 4 Department of Endocrine Oncology and Nuclear Medicine, Gustave Roussy and University Paris-Saclay, 94805 Villejuif, France; livialamartina@gmail.com (L.L.); Martin.SCHLUMBERGER@gustaveroussy.fr (M.S.); 5 Department of Public Health, University of Naples “Federico II”, 80131 Naples, Italy; domenico.salvatore@unina.it

**Keywords:** differentiated thyroid cancer, radioactive iodine therapy, imaging, theragnostic, diagnosis

## Abstract

**Simple Summary:**

Since the 1940s, radioactive iodine has been used for functional imaging and for treating patients with differentiated thyroid cancer (DTC). During this long-lasting experience, the use of iodine isotopes evolved, especially during the last years due to improved knowledge of thyroid cancer biology and improved performances of imaging tools. The present review summarizes recent advances in the field of functional imaging and theragnostic approach of DTC.

**Abstract:**

The present review provides a description of recent advances in the field of functional imaging that takes advantage of the functional characteristics of thyroid neoplastic cells (such as radioiodine uptake and FDG uptake) and theragnostic approach of differentiated thyroid cancer (DTC). Physical and biological characteristics of available radiopharmaceuticals and their use with state-of-the-art technologies for diagnosis, treatment, and follow-up of DTC patients are depicted. Radioactive iodine is used mostly with a therapeutic intent, while PET/CT with ^18^F-FDG emerges as a useful tool in the diagnostic management and complements the use of radioactive iodine. Beyond ^18^F-FDG PET/CT, other tracers including ^124^I, ^18^F-TFB and ^68^Ga-PSMA, and new methods such as PET/MR, might offer new opportunities in selecting patients with DTC for specific imaging modalities or treatments.

## 1. Introduction

Radioactive iodine (RAI) has been used since the 1940s as a specific radiopharmaceutical agent for functional imaging and for treating patients with differentiated thyroid cancer (DTC) [1,2,3,4,5,6]. During this long-lasting experience, the use of RAI (^123^I/^131^I) for imaging [7] and for therapy evolved, especially during the last years. This is related to the improved knowledge of the biology and natural history of thyroid cancer. In fact, a standard initial treatment was advocated for all DTC > 1.5 cm in diameter, consisting of a total thyroidectomy, followed by a post-operative administration of a high activity of ^131^I (>3700 MBq) and then by a thyroid hormone treatment that suppressed the serum TSH [8]. Currently, this has changed toward a more selective approach based on prognostic indicators and on the presence or absence of evidence of persistent disease [4,9].

Theragnostic is a treatment strategy that combines therapeutics with diagnostics. It associates a diagnostic test that identifies patients most likely to be helped or harmed by a targeted therapy and the use of this therapy based on the test results. In metastatic DTC patients, the RAI diagnostic whole-body scan (WBS) will search for foci of uptake to be treated and will quantify uptake for dosimetry and the post-therapy WBS will control that these foci have been treated and may also quantify the radiation dose to each neoplastic focus [10]. This appealing strategy that is followed by many nuclear medicine experts needs to be validated by prospective trials in terms of clinical benefits for the patients.

Another area of remarkable progress is the improvement in performances of scintigraphy devices with hybrid imaging implementation both for single photon emission tomography (SPECT) and positron emission tomography (PET) techniques [11]. The integration of SPECT with computed tomography (CT) in SPECT/CT tools improved the quality and accuracy of ^131^I imaging and allows a quantitation of any uptake and potentially a lesion dosimetry evaluation before and after RAI treatment. PET/CT has become an integral part of DTC management using the non-specific ^18^F-fluorodeoxyglucose (^18^F-FDG) tracer both for diagnostic and prognostic purposes and, where available, using ^124^I that is a highly performing radiopharmaceutical to evaluate in vivo iodine avid lesions [12,13]. Unfortunately, most evidence concerning the use of these tracers relies on retrospective data or on expert opinion or consensus statements. Only few prospective studies that provide unbiased data are available and are detailed in the present review that summarizes recent advances in the field of functional imaging and theragnostic approach of DTC.

## 2. Natural History of Differentiated Thyroid Cancer

There are four histological types of differentiated thyroid cancer: papillary the most frequent, follicular, oncocytic (or Hürthle) and poorly differentiated [14,15]. The TNM classification evaluates the risk of thyroid cancer death [16], and the American Thyroid Association (ATA) risk stratification evaluates the risk of structural recurrence [4]. Most thyroid cancers (>80%) are at low risk of death and of recurrence and only require limited treatment and limited follow-up modalities.

Papillary thyroid cancer spreads to neck lymph nodes and then to lungs; lung metastases are usually multiple, diffuse and frequently with a miliary aspect. Follicular and oncocytic cancers spread to lungs and bones, but lymph node involvement is less frequent than in papillary thyroid cancer. The other organs (brain and liver) are less frequently involved. Currently, distant metastases are observed in less than 5% of all thyroid cancer patients and are mostly observed in high-risk patients [17,18].

Differentiated thyroid cancer produces thyroglobulin (Tg) that is used as a specific and sensitive serum marker of the disease [4,9]. Interferences due to the presence of serum Tg antibodies should be excluded because they may lead to false negative results and less frequently to false positive results. After total ablation of the thyroid gland with surgery and post-operative administration of RAI, serum Tg should be undetectable and any detectable Tg signals the persistence of thyroid cells. After surgery only, remnants of normal thyroid tissue may produce detectable amounts of Tg in the serum; however, following total thyroidectomy the size of these remnants is normally small and serum Tg is frequently undetectable or detectable at a low level on thyroid hormone treatment. False negative serum Tg measurements are rare and mostly observed in patients with small tumor foci in neck lymph nodes or in lungs.

Functional imaging is a sensitive tool for locating a residual thyroid cancer focus and can also provide prognostic information. RAI uptake in neoplastic foci is associated with the efficacy of RAI treatment and with a better prognosis, while FDG uptake on PET/CT can be associated with a more aggressive behavior and RAI refractoriness [4].

## 3. Radiopharmaceuticals for DTC Patients

### 3.1. Available Radioactive Isotopes of Iodine for Clinical Use Include ^123^I, ^131^I and ^124^I

The metabolism of RAI in thyroid cells depends on several steps, including iodine uptake and organification that are altered in thyroid cancer tissues, resulting in a low iodine concentration and in a short effective half-life of iodine in the cells compared to normal thyroid tissue [5]. These abnormalities are related to a strongly reduced expression of the Natrium/Iodide Symporter (NIS) and of thyroid peroxidase (TPO) mRNA, but Tg, SLC26A4 (which encodes pendrin), Dual oxidase (Duox) and thyroid stimulating hormone receptor (TSH-R) gene expression is still present in most differentiated thyroid cancers [6,19]. The TTF-1, FOXE1, PAX-8 and HEX transcription factors are implicated in thyroid development 19. Altered expression levels of TTF-1 and PAX-8, have been demonstrated in DTC [20], and NIS expression levels were significantly related to PAX-8 and to HEX expression levels. Immunohistochemistry confirmed that NIS protein expression is profoundly decreased in differentiated thyroid cancer tissues, and in positive samples, NIS protein is detected in only a few malignant cells that appear to be polarized [21]. Other studies suggested that in some thyroid cancer tissues the NIS protein is present in the intra-cellular compartments but is not transported to the cell membrane, and this may explain why it is not biologically active [22]. TPO biological activity is reduced [23] and TPO immunostaining is weak or absent in most carcinomas when using the monoclonal antibody MoAb47 [24]. 

During thyroid hormone treatment in the absence of TSH stimulation there is no detectable uptake of RAI in neoplastic lesions.

Following TSH stimulation, RAI uptake is detectable in 70% of patients with metastases and serum Tg level increases in nearly all patients with metastases [25,26]. These data clearly show that TSH stimulates functional parameters of thyroid cancer cells

DTC are characterized by several genomic alterations [27]. In particular, the BRAF V600E point mutation is the most frequent alteration in papillary thyroid cancers (PTC) (45–60%); RAS and then TERT promoter point mutations can be also found and gene fusions (TRK 1/3, RET, ALK, BRAF) are detectable in only 15% of tumor samples [28]. These alterations result in an aberrant activation of the MAPK pathway that is found in 85% of PTC and has a crucial role in the impairment of iodide uptake and metabolism. RAS, TERT promoter, TP53, EIF1AX, PTEN, RB1, GNAS point mutations and PAX8-PPARG, ALK, NTRK 1/3, RET fusions can be detected in follicular thyroid carcinomas [29,30]. The spectrum of mutations that can be found in Hürthle cell carcinoma is slightly different [31]. The density of mutations is higher in poorly differentiated thyroid cancers, BRAF and RAS point mutations being the most frequent alterations [32].

In pre-clinical models of DTC, a forced expression of the BRAF V600E mutated gene in thyroid cells impaired the expression of almost all functional genes, which could be restored by ceasing its expression or by suppressing the MAPK pathway activity with a BRAF or a MEK inhibitor [33,34]. PTC patients with a BRAF V600E mutation demonstrate decreased or absent expression of thyroid functional genes coding for NIS and TPO [35,36]. The presence of a TERT promoter mutation is associated with a poor outcome [27,28]. Of note, while about 30% of all DTC patients with metastases do not demonstrate any RAI uptake following TSH stimulation [9], this proportion increases to 70% in those with BRAF V600E mutation and to 97% in presence of both BRAF V600E and TERT promoter mutations [37]. In conclusion, PTC classified as BRAF-like tumors are more dedifferentiated than RAS-like PTC, and metastases with RAI uptake are enriched in RAS mutated tumors [38]. Furthermore, in BRAF V600E mutated tumors besides the decrease in iodine gene expression there is an increase in the Glucose Transporter-1 (GLUT-1) gene expression [39] (see Section 3.4) indicating a more aggressive disease [40].

### 3.2. Physical Characteristics of Radioactive Iodine Isotopes

Physical characteristics of radioactive iodine isotopes are summarized in Table 1.

^123^I has a physical half-life of 13 hours and decays by electron capture with emission of a photon with an energy of 159 keV allowing good imaging quality. However, its high cost and limited availability make its clinical use limited in routine practice [41]. ^131^I is characterized by a physical half-life of 8.02 days. From its decay beta particles are emitted with a maximal energy of 606 keV and a mean energy of 191 keV and gamma rays, with a main gamma ray energy of 364 keV that make this radioisotope suitable for both therapeutic purpose and imaging. Beta particles can cause multiple ionizations before losing their energy and are responsible for most of the therapeutic effect. The penetration of electrons in soft tissue is about 1 mm and their damaging effect is restricted to a large extent to thyroid cells. The detection of gamma rays allows diagnostic imaging after low activity administration and post-therapeutic imaging after the administration of a high activity [42]. This detection can also be used for dosimetry purposes. ^131^I is cheaper than the other radioactive iodine isotopes and is widely available. ^124^I is a positron emitting isotope suitable for PET imaging. Its half-life is 4.2 days with a complex decay scheme and approximately 23% of the disintegrations result in positron emissions. The main clinical application of ^124^I is diagnostic imaging [3] and lesion dosimetry [43,44,45,46,47]. Unfortunately, ^124^I is expensive, not available in all countries and is mostly used for research purposes.

Thyroid-cancer tissue concentrates RAI only following TSH stimulation. Elevated serum TSH concentrations can be obtained by withdrawing thyroxine for three to six weeks [4,9]. The resulting hypothyroidism can be attenuated by replacing the thyroxine with triiodothyronine (that is more rapidly metabolized) for 3 weeks (25 µg/day during the first week, 50 µg/day during the second week, 75 µg/day during the third week), and then withdrawing it for 2 weeks. Various protocols are used for this preparation to RAI administration and to assure a significant uptake of ^131^I the serum TSH concentration should be higher than 30 mIU/L but the optimal TSH level to be attained is not well defined [4,48]. An alternative method for TSH stimulation of thyroid tissue is the use of recombinant human TSH (rhTSH) that is given intramuscularly (0.9mg) (Thyrogen, Sanofi-Genzyme, Cambridge, USA) for two consecutive days and RAI is administered on the day following the second injection. RhTSH stimulation is performed during thyroid hormone treatment. It is well tolerated; its use avoids hypothyroid symptoms and maintains the quality of life [49]. Clinical trials have demonstrated an efficacy of rhTSH similar to thyroid hormone withdrawal both for diagnostic use during follow-up with serum Tg determination and ^131^I diagnostic whole-body scan (d-WBS) [50] and for post-operative administration of ^131^I [51,52,53]; however, in patients with distant metastases, metastatic RAI uptake was lower following rhTSH than following thyroid hormone withdrawal [54,55].

When RAI administration is planned iodine-containing medications and iodine-rich foods should be avoided, and a delay of 4 weeks should be observed after an injection of contrast medium for CT [56]. In women of childbearing age, pregnancy must be excluded, and RAI should not be administered in breastfeeding women [4,48,57].

### 3.3. Methods Used for Functional Imaging with 131I: WBS and SPECT/CT

For diagnostic ^131^I WBS, 74 to 185 MBq (2 to 5 mCi) of ^131^I is normally given to obtain images with adequate quality, while the recommended activity following rhTSH stimulation is 148 MBq (4 mCi) [50]. Higher activities may reduce the uptake of a subsequent therapeutic activity of ^131^I; this phenomenon is called stunning, but its relevance is still controversial and probably does not contraindicate pre-therapy RAI d-WBS in patients for whom it might be beneficial [58,59]. Scanning is performed 2 to 3 days after the administration, using a double-head whole-body gamma camera equipped for the high energy of the main photon emitted by ^131^I (364 keV) with thick crystals and high-energy collimators.

Fractional uptake in neoplastic foci is assumed to be equivalent after the administration of a low or a high activity of ^131^I, and low uptake that may be no detectable after 74 to 185 MBq (2 to 5 mCi) and may be detectable only after the administration of 3700 MBq (100 mCi) or more. This is the rationale for performing a WBS 3 to 7 days after each therapeutic administration of ^131^I, that will also control and quantitate the RAI uptake in neoplastic foci [60].

Accurate anatomic localization of all foci of uptake is feasible with the use of SPECT/CT that ensures differentiation between uptake in lymph nodes and that in normal thyroid remnants in the neck, in ribs or in lungs in the chest, in the urinary or digestive tract or in bones in the pelvis [61,62,63]. Both pre-therapeutic diagnostic and post-therapeutic WBS can be associated with SPECT/CT that also permits a quantitation of uptake and a dosimetry evaluation.

Patient education is mandatory before any ^131^I administration to attenuate body radiation exposure, for image quality improvement and for reduction in false positive images. Thus, lemon juice is suggested to limit salivary gland uptake, ingestion of large quantities of liquid and laxatives may decrease bladder, gonad and colon irradiation. Stimulation of saliva flow with lemon juice given right after ^131^I administration is associated with an increased risk of alterations of salivary gland function [64]. Thus, lemon juice should be given after 24 h following ^131^I therapy. Finally, patients are invited to take a shower and to wear clean clothes before scanning. False positive results are infrequent [65] and are mostly observed on high activity ^131^I WBS.

Even in the presence of high serum TSH concentration, and in the absence of any iodine contamination, only two-thirds of patients with metastatic disease exhibit some ^131^I uptake. Uptake is more frequently observed and is more important in young patients with small metastases from a well-differentiated thyroid tumor [66].

### 3.4. ^18^F-FDG

^18^F-FDG is the most commonly used PET radiotracer for metabolic characterization, detection of recurrence, staging and evaluation of response to therapy especially in most aggressive or less differentiated tumors [67,68]. It is entrapped into cells through glucose transporters (GLUT). The expression of GLUT genes is increased in aggressive differentiated and dedifferentiated thyroid tumors and is related to TSH levels [69]. ^18^F-FDG PET/CT can be performed while the patient is on thyroid hormone treatment, but ^18^F-FDG uptake in neoplastic foci will increase following thyroid hormone treatment withdrawal or following injections of rhTSH, thus slightly increasing both its specificity and sensitivity, but with limited clinical benefits d. Semi-quantitation of uptake is performed using various parameters, including Standardized Uptake Value (SUV) [70].

**Figure 1:**
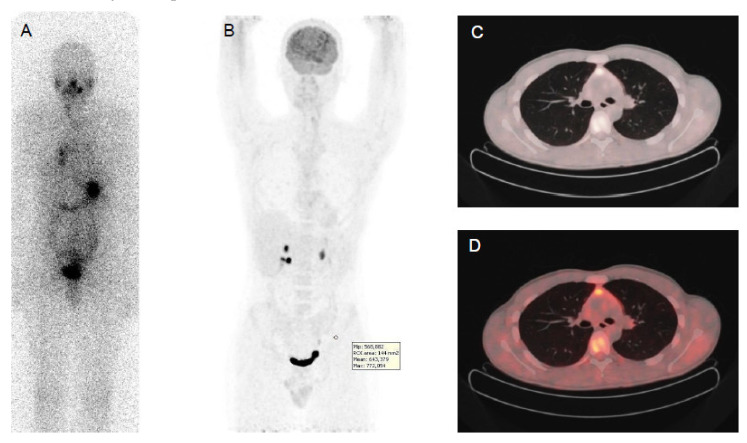
Papillary thyroid cancer in a 41-year-old man with a high thyroid stimulating hormone stimulated serum thyroglobulin level (475 ng/mL). Post-therapy (**A**) ^131^I whole-body scan showed pulmonary uptake and uptake and in neck lymph nodes. ^18^F-FDG PET-CT (**B,C,D**): CT images evidenced multiple small millimetric lung metastases (**B,C,D**), without significant ^18^F-FDG uptake.

The sensitivity of ^18^F-FDG PET/CT increases with higher serum Tg levels and with larger size of the tumor foci. Nevertheless, in case of highly proliferating disease and in those with a short Tg doubling time, the sensitivity can be very high, even in the presence of low Tg levels [71]. ^18^F-FDG uptake can be detected in neck lymph nodes, even in nodes of less than 1 cm in diameter, in lung and in bone metastases. However, PET scan failed to detect abnormal ^18^F-FDG uptake in small miliary lung metastases detected by CT scan, due to an absence of ^18^F-FDG uptake in these well differentiated and slow growing neoplastic cells, and partial volume effect, consisting of the combined effect of poor spatial resolution and the contamination of activity from neighboring tissues or spillover effect (Figure 1) [72,73].

**Figure 2:**
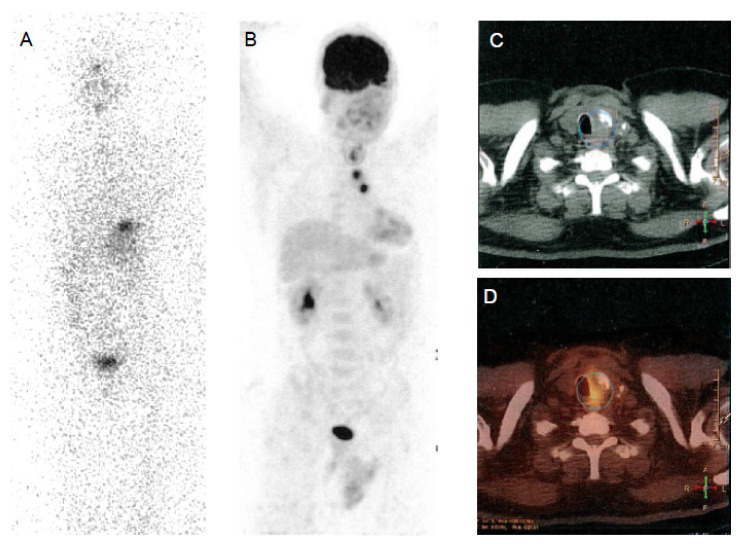
Papillary thyroid cancer in a 30-year-old woman. Post-therapy (**A**) ^131^I whole-body scan and ^18^F-FDG PET-CT (**B,C,D**) images. Post-therapy ^131^I whole-body scan did not show areas of uptake (**A**). ^18^F-FDG PET-CT images evidenced multiple areas of increased uptake in the left retrosternal and latero-cervical region.

In DTC patients, the “flip-flop phenomenon” consists of a mismatch between high glucose and low iodine uptake, due to the elevated glucose metabolism coupled to the dedifferentiation process and has an important prognostic significance (Figure 2). This is observed in patients with iodine refractory thyroid cancer, with poorly differentiated, Hürthle cell cancer, PTC with aggressive features (tall cell variant) or with the BRAF V600E mutation and less frequently in patients with well differentiated papillary or follicular carcinoma. This indicates clinically more aggressive lesions [74,75,76] and the presence of ^18^F-FDG uptake in lesions with also radioiodine uptake indicates that these lesions are less likely to respond to radioiodine treatment [77].

### 3.5. Other Tracers

The ^18^F-tetrafluoroborate (^18^F-TFB) is an analog of iodine that can be used for imaging the NIS expressing cells but that will not undergo organification in the thyroid cells, that is decreased or non-existent in thyroid cancer cells. ^18^F-TFB biodistribution in patients with thyroid cancer is similar to that of ^99m^Tc-pertechnetate [78]. The potential ^18^F-TFB advantages come from the possibility of using a more rapid synthesis of this PET tracer over the less available and more costly ^124^I. In small series of patients, results obtained with ^18^F-TFB appeared similar to those obtained with ^124^I PET/CT [79] or with ^131^I d-WBS [80].

**Figure 3:**
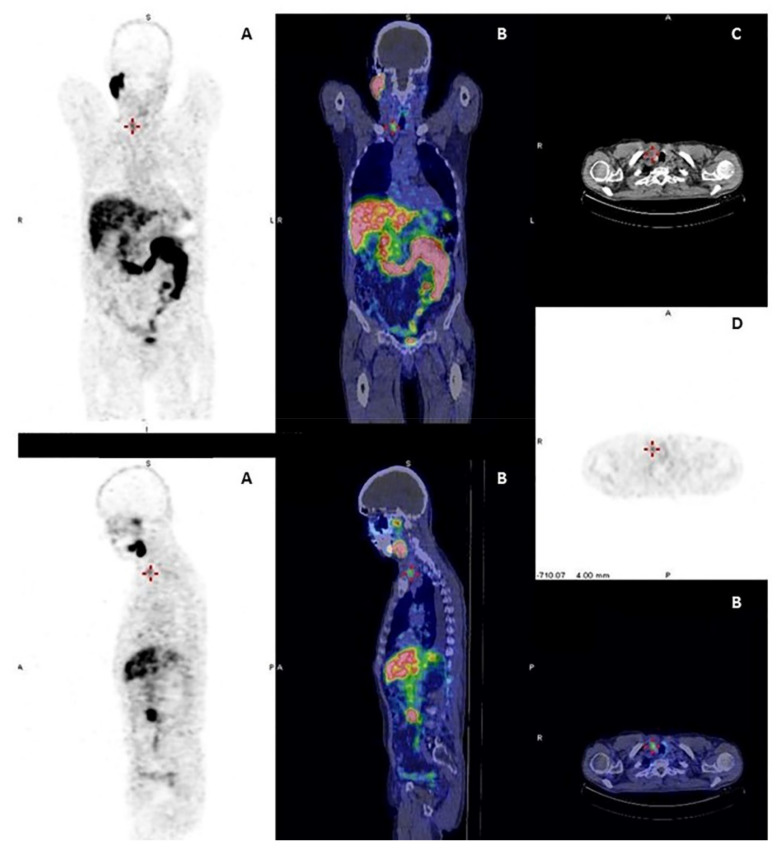
A 76-year-old patient with prostate cancer and a right thyroid nodule of 15 mm in diameter. Multiple image projections (**A**), fusion PET-CT images (**B**), CT images (**C**) and PET images (**D**), respectively, of ^68^Ga-PSMA imaging with high-intensity PSMA accumulation. Fine needle aspiration biopsy of PSMA-avid thyroid lesion was performed revealing an indeterminate cytology. Final histology revealed papillary thyroid cancer.

The prostate-specific membrane antigen (PSMA) is a transmembrane glycoprotein receptor that is highly expressed in prostate carcinoma cells. However, its expression has been associated with neovascular development in several tumors, including DTC [81]. Its expression seems related with a lower differentiation level and more aggressive histology or refractoriness. The ^68^Ga-PSMA was used in small series of DTC patients with some promising results as a theragnostic radiopharmaceutical, giving the possibility in case of positive imaging results to select DTC patients for 177-Lutetium (^177^Lu)-PSMA therapy [82]. Recently, two patients underwent (177Lu)-PSMA therapy and achieved a minor tumor response [82]. The agent 2-(3- {1-carboxy-5- [(6- [(18)F] fuoropyridine-3-carbonyl)-amino]-pentyl}-ureido)-pentanedioic acid (DCPyl) labeled with ^18^F is another PSMA-PET imaging agent with the advantage of imaging obtained with ^18^F consisting in a potential widely available and easily low cost produced tracer (Figure 3) [43]. ^18^F-Fluoride has been used for evaluating with PET patients with bone metastases from thyroid cancer in small series of patients, but these lesions are lytic, frequently with a low uptake and this makes their evaluation difficult [83]. This is in accordance with the absence of efficacy of ^223^Radium in treating bone metastases from DTC [84]. Further studies are needed for evaluating the utility of these tracers in DTC patients.

## 4. PET Imaging Methods

### 4.1. PET/CT

The ring detector system of PET technology benefits of positron emitting radionuclides for detection of coincidences. The positron and electron annihilation results in two 511 keV photons [85,86]. The relatively long half-life of ^18^F (110 minutes) and ^124^I (4.2 days) allows transportation of these radionuclides from the production facility to the imaging sites. The integration of PET stand-alone modality with CT in a single PET/CT device provides several advantages, including a complementary accurate localization and characterization of tumor foci [87]. Moreover, the addition of CT permits attenuation correction. Beyond visual analysis, PET/CT allows semi-quantitative evaluation of tracer uptake, by using several parameters including standardized uptake values (SUV).

### 4.2. PET/MR

Magnetic resonance (MR) imaging is a sensitive diagnostic technique for evaluation of DTC recurrence extent in the neck, mediastinum, bones, and liver. Simultaneous PET/MR provides complementary data. However, high costs and limited spread in diagnostic units limit its clinical routine use. Few studies on limited series of DTC patients found an excellent correlation between the two imaging techniques (PET/MR vs. PET/CT) despite, as expected, an inferior sensitivity of MR in the detection of lung metastases [88]. In a recent work, 40 consecutive DTC patients were evaluated at follow-up by sequential ^18^F-FDG PET/CT and ^18^F-FDG PET/MR imaging scans [89]. ^18^F-FDG PET/MR was positive in 11 patients detecting 33 lesions, while 10 patients showed positive findings with 30 lesions detected at ^18^F-FDG PET/CT.

**Figure 4:**
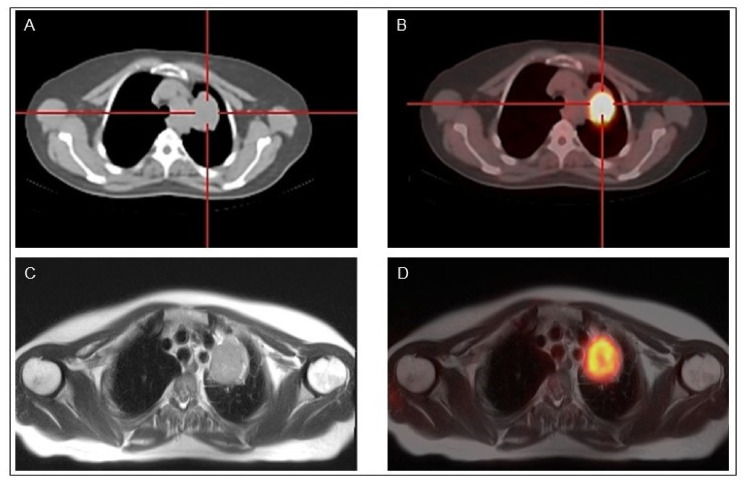
Classical papillary thyroid cancer in a 45-year-old man^18^F-FDG PET-CT (**A,B**) and ^18^F-FDG PET-MR (**C,D**) show a mediastinal lymph node with high ^18^F-FDG uptake.

Indeed, PET/MR when available might be performed in selected patients with bone, soft tissue or liver lesions for whom a MR imaging is indicated (Figure 4).

## 5. Indications for the Use of Radioactive Iodine

Radioiodine exposure has raised major concerns. The radiation dose delivered to the bone marrow after the administration of 3700 MBq in hypothyroid condition was estimated at 0.50 Gy [90]. However, despite reassuring data demonstrating the absence of untoward pregnancy outcome and the low risk of second primary malignancies observed only following the administration of high cumulative activities of radioiodine [91,92,93], the administered activities should be reduced as much as possible.

### 5.1. Post-Operative Administration of RAI

**Figure 5:**
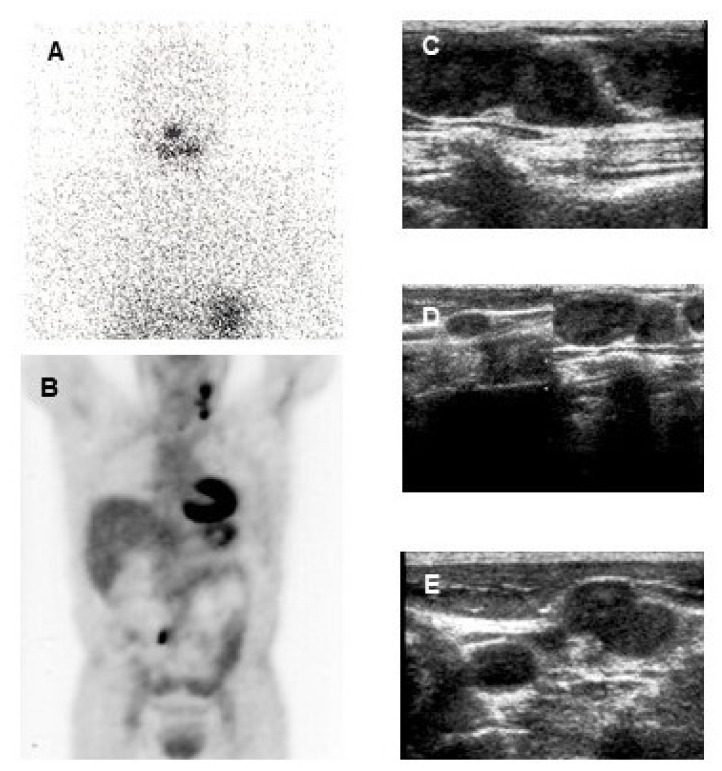
37-year-old woman with a papillary thyroid cancer (tall cell variant) with a post-operative TSH-stimulated serum thyroglobulin (Tg) level of 230 ng/mL. Post-therapy ^131^I whole-body scan (**A**) did not show any area of increased uptake. ^18^F-FDG PET-CT (**B**) evidenced multiple areas of increased uptake in the left latero-cervical region and homogeneous cardiac ^18^F-FDG uptake (that is not an infrequent finding, also in patients without overt cardiac disease). Ultrasound (**C,D,E**) evidenced multiple suspicious lymph-nodes in the left latero-cervical region, with round shape, loss of echogenic hilus and micro lobulated margins. At fine needle aspiration cytology confirmed lymph node metastases from the papillary thyroid cancer and the Tg concentration was elevated (3800 ng/mL) in the aspirate fluid.

In low-risk patients, retrospective studies did not demonstrate consistent benefits of the post-operative ^131^I administration in terms of recurrence and mortality [4,9,94,95,96,97,98]. Discrepant results are, at least in part, related to differences in risk assessment: the TNM classification of the risk of cancer related death was used in many studies to predict the risk of recurrence and a retrospective study based on the ATA risk stratification of recurrence did not show any benefit of the post-operative administration of ^131^I in low-risk patients [98]. This is not surprising given a risk of persistent structural disease after surgery of less than 5% that can be frequently cured with a later treatment and a risk of mortality of less than 1%. Additionally, after total thyroidectomy that normally leaves only small remnants of non-tumoral thyroid tissue, the follow-up can reliably be based on serum Tg determination obtained on thyroid hormone treatment and neck ultrasound, even in the absence of post-operative ^131^I administration (Figure 5) [4,9].

The possibility that post-operative ^131^I might be administered only in a selected subset of low-risk patients derives from several studies and should consider the following points:

- The current definition of excellent response to treatment (and of complete ablation in case of post-operative administration of ^131^I) at 6–12 months is based, in the absence of a control radioiodine WBS [99,100], on undetectable serum Tg level in the absence of Tg-Ab (either < 1ng/mL following rhTSH when using a traditional assay or < 0.2 ng/mL on L-T4 treatment when using a sensitive assay) and a neck ultrasound (US) without any abnormal findings [4,9,101].
- In two prospective randomized non-inferiority trials in low-risk patients, the percentage of complete ablation was similar when using for preparation either rhTSH injections on L-T4 treatment or withdrawal of thyroid hormone treatment and administration of either 1100 or 3700 MBq ^131^I [52,53]. Indeed, with rhTSH there was no hypothyroid symptom, and the quality of life was maintained [49]. With a 5-year follow-up, only few patients (<5%) received further treatments and the same favorable outcome was observed regardless of the initial ablation protocol used [102,103]. In conclusion, when indicated in low-risk patients a post-operative administration of ^131^I should consist in the administration of 1100 MBq following rhTSH injections.
- Less than 5% of these low-risk patients had persistent disease, and this suggests that ^131^I might be not beneficial and may represent over-treatment in the other 95% [4,47]; low-risk patients might be selected for ablation on the basis of the post-operative neck US and serum Tg level, the risk of persistent disease being low in patients with undetectable Tg and increases with higher serum Tg levels [101,102,103,104]. It is, however, still unclear whether the post-operative ^131^I administration may improve the outcome of these patients with detectable post-operative Tg levels. This is being evaluated within two ongoing prospective randomized non-inferiority trials (ESTIMABL2 and ION) that compare the outcome of low-risk patients treated either with 1100 MBq following rhTSH or followed-up with no post-operative ^131^I.

Intermediate-risk patients represent a heterogeneous group of patients, and their management is still under debate. RAI treatment should be planned according to risk factors and post-operative findings [105]. A longitudinal and observational study compared the outcomes of low and lower-intermediate risk (minimal extra-thyroid extension or ≤5 central compartment lymph node metastases) DTC patients undergoing either systematic or selective use of RAI treatment [106]. After 3 years of follow-up, the rate of structural incomplete response was low (1–3%) and did not differ with either approach. After 1 year of follow-up, a significantly higher rate of patients with residual detectable serum Tg was observed after selective RAI as compared to the systematic RAI cohort. Interestingly, this difference was no more statistically significant after a longer follow-up. These data suggest that selective use of RAI permits to achieve comparable low rates of structural disease but poses the issue of the management of persistent residual Tg or Tg-Ab. It is likely that intermediate risk patients with a post-operative low or undetectable serum Tg level have a low risk of persistent disease and can probably be managed such as low-risk patients, but prospective trials in well-defined groups of patients are needed such as the INTERMEDIATE trial currently ongoing in France.

In high-risk patients, high ^131^I activities (3700 MBq or even more) should be administered post-operatively following withdrawal of thyroid hormone treatment [9]. Radioactive iodine in this case aims at ablation of normal thyroid remnants and at eradication of persistent neoplastic foci that may be occult (adjuvant treatment) or known (treatment) with a reported positive impact on patient outcome [107,108].

The ASTRA phase III study (NCT01843062) in high-risk DTC patients failed to demonstrate an improved disease-free survival with selumetinib (a MEK inhibitor)-enhanced adjuvant RAI administration compared with placebo and adjuvant RAI. This may be due to the low inhibitory efficacy of selumetinib. As already pointed out, in patients with a BRAF V600E mutation, in particular if a TERT mutation is also present, the RAI uptake in the neoplastic tissue is frequently absent, and these patients might benefit from a redifferentiation program before being treated with RAI, but further trials are needed (see Section 7).

### 5.2. Use of Radioiodine for Diagnosis

In the past, ^131^I d-WBS was routinely used in many situations, post-operatively to assess the completeness of surgical resection and the potential indication for the administration of a high activity of radioiodine, during follow-up to assess excellent response or to detect persistent and recurrent disease and before any therapeutic administration for persistent or recurrent disease to assess the presence of radioiodine uptake in neoplastic foci. At the present time, d-WBS is less frequently performed: post-operative administration of ^131^I is mostly based on the surgical report, on prognostic indicators and on post-operative serum Tg level and neck US findings; additionally, the completeness of total thyroidectomy is improved, leaving in most patients only small remnants of non-tumoral thyroid tissue and in this condition the post-therapy WBS is frequently more informative than the d-WBS that is currently not routinely performed in many centers in low risk patients. A recent statement proposed that a d-WBS might be selectively performed in intermediate risk patients in the post-operative staging to guide, in addition to clinical and surgical data the decision of a therapeutic RAI administration and select a personalized patient-based activity [105]; however, in the absence of prospective data, the benefits afforded by the d-WBS remain uncertain. A randomized trial in lower-intermediate risk DTC patients (estimated risk of recurrence about 8%) comparing systematic RAI administration (3700 MBq) to a selective RAI administration based on post-operative rhTSH stimulated Tg, neck US and RAI d-WBS is ongoing in France (INTERMEDIATE trial, NCT04290663).

Follow-up is currently based on the combination of serum Tg determination and of neck US and when performed, ^131^I d-WBS does not improve the detection of persistent/recurrent disease in the neck and control d-WBS is not indicated in patients with undetectable serum Tg and neck US with no abnormal finding [99,100,101]; control d-WBS may be indicated in patients with large thyroid remnants in whom the post-therapy WBS was poorly informative, in those with equivocal foci of uptake on the post-therapy WBS, and in high risk patients with detectable serum Tg-Ab in whom serum Tg determination is not reliable [4].

## 6. Comparison between ^131^I and ^124^I

^124^I PET/CT imaging offers several benefits, including improved spatial resolution and better diagnostic sensitivity, over ^131^I SPECT imaging [109]. ^124^I PET scanning can be performed up to 120 hours after the tracer administration. The sensitivity of ^124^I PET/CT in identifying DTC lesions was excellent (94.2%), being better than ^123^I/^131^I d-WBS, and at least equivalent to post-therapy ^131^I WBS planar imaging and even demonstrates more lesions in some patients [43]. In DTC patients, ^124^I can also be used for the pre-therapeutic lesion dosimetry.

## 7. Use of Radioactive Iodine for Treatment of Distant Metastases

Treatment of distant metastases with RAI is based on the administration of high ^131^I activities [110,111,112]. A relationship between the radiation dose delivered to tumor foci and its efficacy has been reported. The mean recommended radiation dose to treat metastases is 100 Gy [113,114] and the clinical issue is to administer an activity of ^131^I high enough to deliver an effective radiation dose to tumor foci. The absorbed dose depends on the initial radioactive concentration of ^131^I in the tissue, namely the ratio between the initial total uptake and the mass of neoplastic tissue and on its effective half-life in the tissue, i.e., the time after which the radioactivity in the tissue has decreased by a factor of 2. The effective half-life is related to both ^131^I physical half-life (8.02 days) and to its biological half-life, which is related to its elimination from the tissue. Even though the administered activity of ^131^I is high, the absorbed dose delivered to neoplastic foci might be suboptimal for successful therapy due to a low radioiodine concentration and to a decreased organification rate resulting in a short effective half-life of RAI in the lesion. While iodine uptake is about 1%/g of the administered activity in normal thyroid tissues, it ranges from 0.1% to 0.001%, or is even lower in neoplastic tissues and the effective half-life of ^131^I ranges from 6–8 days in normal thyroid tissues and only 2–5 days or even less in neoplastic tissues [5]. Attempts have been made to improve RAI efficacy, including prolonged withdrawal of thyroid hormone treatment that stimulates higher metastatic uptake than rhTSH injections [54,55], avoiding iodine contamination and optimizing the administered activity of ^131^I. The administered activity can be standard or determined by pre-therapeutic dosimetry [44,45,46,47,115]. Pre-therapeutic lesion dosimetry from repeated three-dimensional imaging with ^131^I SPECT/CT or ^124^I PET/CT after a tracer administration enables reconstruction of the time–activity curve within the lesion and will permit to determine the activity to be administered to deliver an optimal radiation dose to the lesion. The volume in which uptake occurs can be estimated with the current use of three-dimensional imaging, using ^131^I and SPECT/CT. The poor spatial resolution of this imaging technique is a limiting factor for accurate measurement of small volumes. Moreover, the absolute quantitation of ^131^I concentration in small lesions is challenging because of the complex detection and image reconstruction processes [116]. PET/CT using ^124^I provides a higher spatial resolution and a semi-quantitative measurement of radioactivity [44,45,46,47]. 

Whole-body/blood clearance dosimetry determines the maximal activity that can be administered so that the absorbed dose to blood does not exceed 200 cGy and in the presence of iodine-avid diffuse lung metastases that the whole-body retention does not exceed 2960 MBq (80 mCi) at 48 h after administration, in order to prevent toxic effects, respectively, on the bone marrow and lungs [117,118,119,120]. 

A retrospective study showed a similar overall survival of DTC patients with distant metastases treated either with a standard activity of 3700 MBq or with higher activities determined with blood dosimetry [115]. This investigation is in accordance with other studies that found no evidence that pre-therapeutic dosimetry might improve the outcome of metastatic patients [44,45,46,47]. Indeed, prospective studies are needed in metastatic patients to define the role and the impact of dosimetry. 

Between ^131^I treatment courses, thyroid hormone treatment decreases serum TSH to low or undetectable levels. Complete tumor responses were observed in one third of patients with distant metastases, mostly in young patients with small metastases from a well differentiated cancer, with initial high ^131^I uptake [66]. Unfortunately, not all metastases with ^131^I uptake respond to ^131^I treatment, and this is frequently the case of large metastases, elderly patients or in the presence of high ^18^F-FDG uptake on PET/CT scan [77]. Several explanations have been proposed [5]: the radiation dose might be not high enough to induce cell killing, but as already mentioned increasing the administered activity may not increase enough the dose to the tumor to improve anti-tumor efficacy; anti-tumor efficacy depends also on the sensitivity of thyroid cancer cells to radiation and this is not routinely taken into account; tumor heterogeneity with radiation doses that may differ from one focus to another [44] and also heterogeneous expression of NIS among tumor cells within a single metastasis [5,21]. Finally, the dose estimated by pre-therapy dosimetry with a low activity may differ from the dose effectively delivered during a ^131^I treatment due to a stunning effect induced by the high radiation dose delivered during treatment and this can be assessed by a post-therapy WBS with quantitation of uptake.

When there is no evidence of RAI uptake in the metastases or when ^131^I avid metastases progress despite RAI treatment, the disease is considered ^131^I refractory and RAI is of no further utility becomes useless. This occurs in about two-thirds of patients with extensive disease, who have then a reduced 10-year life expectancy of only ~10%, and who will require other treatment modalities, particularly in case of large tumor burden, symptoms or risk of local complication or documented rapid tumor progression [121]. A redifferentiation program has been used in several trials in patients with advanced refractory thyroid cancer with a RAS or a BRAF mutation [122,123]. The administration for 4–6 weeks of a BRAF or a MEK inhibitor either alone or in combination induced the re-apparition of radioiodine uptake in 40 to more than 60 % of patients that allowed treatment with ^131^I after rhTSH stimulation [124,125,126]. This treatment is also capable of restoring a more differentiated histologic appearance and induces an increased production of Tg and thyroid hormones in the serum. A RECIST partial tumor response was then achieved in 25% up to more than 40% [127]. This strategy may apply also to DTC harboring other molecular abnormalities treated with selective inhibitors, as suggested by the RAI uptake restoration observed in a RAI refractory DTC patient with a NTRK fusion treated with Larotrectinib [128] and in a patient with a RET fusion treated with Selpercatinib [129] An open label multicentric clinical trial is ongoing in France (NCT 03244956) testing the efficacy in terms of objective response rate of an anti-MEK or anti-MEK and anti-BRAF selective inhibition before RAI treatment in metastatic RAI-refractory DTC with, respectively, a RAS or BRAF mutation.

## 8. Use of ^18^F-FDG PET/CT in Clinical Practice

### 8.1. ^18^F-FDG PET/CT for the Post-Operative Staging of Aggressive Disease

Patients with a histologically aggressive disease (Hürthle cell, poorly differentiated, widely invasive follicular carcinoma or with an aggressive variant of papillary thyroid cancer, including tumors harboring a BRAF V600E mutation) may demonstrate less differentiated cancer cells, with low serum Tg and no ^131^I uptake. These patients may be referred to initial ^18^F-FDG PET/CT before any treatment or after surgery when there is a suspicion of persistent disease [75,130], since the TSH-stimulated serum Tg and the ^131^I WBS cannot exclude persistent disease.

### 8.2. ^18^F-FDG PET/CT for Elevated Serum Tg and No Other Evidence of Disease

Patients with detectable serum Tg level on thyroid hormone treatment (>5–10 ng/mL) and no other evidence of disease (i.e., with a biochemically incomplete response) need further imaging modalities, especially when the serum Tg level increases over time. In the past, administration of large empiric activities of ^131^I have been encouraged due to the poor sensitivity of the gamma camera detectors available at that time [131]. Currently, ^18^F-FDG PET/CT is a reliable technique to identify occult neoplastic foci in these patients [70]. In 34 DTC patients with elevated serum Tg that increased over time, ^18^F-FDG PET/CT was more sensitive than ^131^I post-therapy WBS in detecting neoplastic foci (88% vs. 16% of 75 foci, respectively) [131]. The frequency of abnormal findings in ^18^F-FDG PET/CT increases with higher serum Tg levels, and this is the rationale for considering ^18^F-FDG PET/CT only in patients with a serum Tg level above 5–10 ng/mL on thyroid hormone treatment or in case of rapid Tg doubling time, independently of its absolute value [71]. As already mentioned, the use of rhTSH stimulation before 18F-FDG PET/CT in patients with low serum Tg and no other evidence of disease slightly increases its sensitivity for the detection of neoplastic foci, but with limited clinical benefits [74]. Additionally, tumors with high ^18^F-FDG uptake generally fail to concentrate ^131^I and in case of detectable ^131^I uptake they frequently fail to respond to ^131^I treatment [77], and thus ^131^I treatment could be less appropriate in these patients. The use of empiric ^131^I therapy could be suggested only in the few patients with serum Tg levels increasing over time and without ^18^F-FDG uptake. 

Furthermore, an overall 84% sensitivity and a 78% specificity of ^18^F-FDG PET/CT has been observed for the detection of recurrent disease in the presence of elevated Tg-Ab levels and negative ^131^I WBS [132].

### 8.3. ^18^F-FDG PET/CT in Patients with Structural Disease

In these patients with structural disease, imaging could visualize neoplastic foci, the extent of disease ranging from small lymph node metastases in the neck to large multiple distant metastases. 

Small lymph node metastases (< 1 cm in diameter) may be treated with ^131^I in the presence of radioiodine uptake and with surgical resection either as first line treatment or in case of persistence after several ^131^I treatment courses [132,133,134]. In patients with a more extended neck recurrence and in those with distant metastases, ^18^F-FDG PET/CT is performed before defining treatment: it provides an extensive work-up of the extent of the disease both in the neck and at distant sites. In patients with metastatic RAI uptake, ^18^F-FDG PET/CT may identify some lesions with low or absent RAI uptake and, therefore, not seen on RAI WBS. This can be complemented with CT scan with contrast medium and/or MRI of the neck and mediastinum that will better define the local extent of the disease. There is usually no ^18^F-FDG uptake in miliary lung metastases that are typically observed in young patients. These miliary lung metastases might be visualized on CT scan, but in some patients because of their small size they are visualized only on post therapeutic RAI WBS. MRI of the liver, bones and brain may also be useful. ^18^F-FDG PET/CT also provides prognostic information, with tumor with high ^18^F-FDG uptake characterized by a more aggressive course [74] and responding less frequently to ^131^I treatment [77]. However, in a given patient, the tumor growth rate of a given metastasis at 1 year is not related to the intensity of its ^18^F-FDG uptake [135]. Semi-quantitative ^18^F-FDG uptake is a parameter used in complement to tumor volume assessment for the follow-up of patients with tumor foci and to help deciding their treatment. ^18^F-FDG PET/CT is also useful for assessing the completeness of focal treatment modalities, such as thermal ablation [74] and the efficacy of systemic treatment using either tyrosine kinase inhibitors or immunotherapy with check point inhibitors [4,9,76].

^18^F-FDG uptake is not specific and false positive results have been reported in up to 39 % of patients [68]. For this reason, a fine needle aspiration biopsy should confirm the thyroid origin of any focus of uptake in neck lymph node areas before defining a therapeutic strategy.

## 9. Conclusions

Functional imaging plays a central role in the management of DTC patients, in particular in those at intermediate or high risk. Radioactive iodine is used in DTC patients mostly with a therapeutic intent, either post-operatively or in patients with known structural disease for whom it is often the first line systemic treatment, and a sensitive WBS is performed a few days after the administration. ^124^I showed encouraging results in a dosimetric approach, but its potential clinical benefit is still not demonstrated.

PET/CT with ^18^F-FDG complements the use of radioactive iodine at the initial evaluation in patients with high-risk DTC, during follow-up in those with rising serum Tg levels over time and in the work-up of patients with documented structural disease. 

## Figures and Tables

**Table 1:** RAI isotopes available for diagnosis and treatment of DTC.

Nuclide	Production	Decay Mode	Energy (keV)	Half-Life	Applications
^123^I	Cyclotron	Electron capture	159	13.22 hours	SPECT imaging
^131^I	Nuclear reactor	β- decay γ decay	606 364	8.02 days	Therapy SPECT imaging Dosimetry
^124^I	Cyclotron	β+ decay	511	4.18 days	PET imaging Dosimetry

## Abbreviations

ATA	American Thyroid Association
CT	Computed tomography
DTC	Differentiated thyroid cancer
^18^F-FDG	^18^F-fluorodeoxyglucose
^18^F-TFB	^18^F-tetrafluoroborate
GLUT	Glucose Transporter
GLUT-1	Glucose Transporter-1
MR	Magnetic resonance
NIS	Natrium/Iodide Symporter
PET	Positron emission tomography
PSMA	Prostate specific membrane antigen
RAI	Radioactive iodine
SPECT	Single photon emission tomography
Tg	Thyroglobulin
TPO	Thyroid peroxidase
TSH	Thyroid stimulating hormone
US	Ultrasound
WBS	Whole-body scan
d-WBS	Diagnostic-WBS
